# IN TIME: PUBLONS SEEKS TO ATTRACT REVIEWERS AND IMPROVE PEER REVIEW

**DOI:** 10.1590/1984-0462/;2017;35;4;00018

**Published:** 2017

**Authors:** Lilian Nassi-Calò

**Affiliations:** a Comunicação Científica em Saúde, BIREME/OPAS/OMS, São Paulo (SP), Brasil.

Peer review, one of the pillars of scholarly communication, has been undergoing a transition moment toward greater reliability, transparency and accountability.^1^

The saturation through which the current model of peer review is going through is a well-known fact to the academic community. The continuous increased amount of publications is not accompanied by the growing number of researchers who referee the submitted articles. As a result, the quality of the reviews has diminished and the time to obtain them, on the other hand, has increased considerably, causing unwarranted delays in the dissemination of knowledge and the advancement of science.

Scientific societies, research institutions, funding agencies and researchers have been devoting themselves to studying the theme; conferences and workshops are organized around the world to discuss new proposals and the future of peer review,^2^ ^,^ ^3^ ^,^ ^4^ including the possibility of prescinding from it, at least in parts.^5^ New forms of assessment emerged, in addition to the classic simple and double-blind models, such as the cascade and post-publication evaluation. This last modality, particularly, has been adopted by a growing number of journals, in addition to preprints repositories.^6^

Peer review is an extremely specialized work which demands time and knowledge, for which the referee - at least until recently - does not receive credits in proportion to their efforts. Most of the time, this painstaking work is discarded after the manuscript is accepted for publication. On the other hand, there is also no adequate training to teach reviewers. Researchers often learn the work alone, or with the help of their mentors, peers, or supervisors throughout their careers. It is not surprising, therefore, the difficulty in obtaining good reviews within the time advocated by the journals’ editorial process.

How to make the task of reviewing articles more appealing to researchers? An initiative created in New Zealand in 2012 by Andrew Preston and Daniel Johnston intends to answer this question. Publons^7^ was launched as startup, initially to register the contribution of referees in a Web portal and to encourage researchers to post online their experiences as peer reviewers. Its creators had their mind set to stimulate researchers to act as referees by offering a platform for recording this activity. In their opinion, being selected by an editor to review an article is a proof of recognition as a specialist in the field, and this fact deserves to be recorded and highlighted. Moreover, they expect that the mechanisms of evaluation of academic performance and reward start recognizing the referees’ work with due relevance in the analysis of curricula.

Operating the Publons portal is quite simple and consists of creating a login, by the researcher, who registers on their personal page their activity as a pre- or post-publication peer reviewer, informing the name of the journal, their area of expertise, the year the review was carried out, and whether the review was pre- or post-publication. The reviews themselves are not made available until the journal authorizes their publication in the blind (the name of the reviewers remain confidential), double blind (the name of authors and reviewers remain confidential) or open format. The author of a reviewed article registered on Publons may choose to make his review openly available only if both reviewers and the journal (through its registered policy at Publons, as may be seen in Plos One Publons)^8^ allow so. It is important to note that, even if the reviews are not made available at the Publons portal, the credit to the reviewers is always recorded.

Besides recording the work of referees, Publons aims to contribute to increasing the reliability of science by combating fraud in peer review. It should be noted that the company was recently acquired by Clarivate Analytics - the company that owns Web of Science and other indexing, publishing, and science evaluation products. Thus, by associating citation data from authors with their reviewing records in Publons, it is possible to journal editors to spot potential fictitious referees who cheat the peer review process. In addition, Publons intends to contribute to the formation of peer reviewers through the Publons Academy:^9^ “a practical online peer review free training course for early career researchers developed together with expert academics and editors to teach you the core competencies and skills needed of a peer reviewer”.

The future of peer review in scholarly communication most likely includes a strong component of technological innovation, preprint servers, and post-publication peer review. The saturation of the current pre-publication peer review model requires simple solutions - such as Publons, for example - and recognition by the evaluation mechanisms of academic performance to grant reviewers due credit.
